# Supplementary material for: Cost-effectiveness and budget impact analyses of dengue vaccination in Indonesia
Source: PLoS Negl Trop Dis. 2021 Aug 12;15(8):e0009664. doi: 10.1371/journal.pntd.0009664 (PMC8384188; doi:10.1371/journal.pntd.0009664)
Supplement: S1 Appendix — (PDF) [file pntd.0009664.s001.pdf]

| *age* | *interval* | *mx* | *qx* | *px* | *lx* | *dx* | *Lx* | *Tx* | *ex* | *a(x)* |
|---|---|---|---|---|---|---|---|---|---|---|
| [0,1) | 1 | 0.0230 | 0.0348 | 0.9652 | 100,000 | 3,479 | 97,564 | 6,296,836 | 63.0 | 0.5 |
| [1,5) | 4 | 0.0010 | 0.0106 | 0.9894 | 96,521 | 1,027 | 413,944 | 6,199,272 | 64.2 | 2.0 |
| [5,10) | 5 | 0.0010 | 0.0082 | 0.9918 | 95,494 | 787 | 475,501 | 5,785,328 | 60.6 | 2.5 |
| [10,15) | 5 | 0.0010 | 0.0072 | 0.9928 | 94,707 | 677 | 471,840 | 5,309,827 | 56.1 | 2.5 |
| [15,20) | 5 | 0.0010 | 0.0103 | 0.9897 | 94,029 | 966 | 467,733 | 4,837,987 | 51.5 | 2.5 |
| [20,25) | 5 | 0.0015 | 0.0113 | 0.9887 | 93,064 | 1,048 | 462,700 | 4,370,253 | 47.0 | 2.5 |
| [25,30) | 5 | 0.0015 | 0.0163 | 0.9837 | 92,016 | 1,502 | 456,326 | 3,907,553 | 42.5 | 2.5 |
| [30,35) | 5 | 0.0020 | 0.0180 | 0.9820 | 90,514 | 1,633 | 448,489 | 3,451,228 | 38.1 | 2.5 |
| [35,40) | 5 | 0.0025 | 0.0175 | 0.9825 | 88,881 | 1,553 | 440,526 | 3,002,738 | 33.8 | 2.5 |
| [40,45) | 5 | 0.0035 | 0.0291 | 0.9709 | 87,329 | 2,540 | 430,294 | 2,562,213 | 29.3 | 2.5 |
| [45,50) | 5 | 0.0055 | 0.0452 | 0.9548 | 84,789 | 3,828 | 414,373 | 2,131,918 | 25.1 | 2.5 |
| [50,55) | 5 | 0.0085 | 0.0698 | 0.9302 | 80,960 | 5,650 | 390,677 | 1,717,545 | 21.2 | 2.5 |
| [55,60) | 5 | 0.0130 | 0.0893 | 0.9107 | 75,310 | 6,722 | 359,746 | 1,326,868 | 17.6 | 2.5 |
| [60,65) | 5 | 0.0220 | 0.1660 | 0.8340 | 68,588 | 11,387 | 314,473 | 967,122 | 14.1 | 2.5 |
| [65,70) | 5 | 0.0345 | 0.2045 | 0.7955 | 57,201 | 11,699 | 256,757 | 652,649 | 11.4 | 2.5 |
| [70,75) | 5 | 0.0550 | 0.3234 | 0.6766 | 45,502 | 14,715 | 190,722 | 395,892 | 8.7 | 2.5 |
| [75,+) | 5 | 0.0895 | 1.0000 | 0.0000 | 30,787 | 30,787 | 205,171 | 205,171 | 6.7 | 2.5 |

| *Age (years)* | | *Cohort* | *Mortality* | *Survival* | *Life Exp.* |
|---|---|---|---|---|---|
| 9 | - | 4,701,100 | 0.0002 | 1.0000 | 62.6141 |
| 9 | 0.5 | 4,700,630 | 0.0002 | 0.9999 | 62.1262 |
| 10 | 1 | 4,700,160 | 0.0002 | 0.9998 | 61.6383 |
| 11 | 2 | 4,699,220 | 0.0002 | 0.9996 | 60.6619 |
| 12 | 3 | 4,698,280 | 0.0002 | 0.9994 | 59.6852 |
| 13 | 4 | 4,697,341 | 0.0002 | 0.9992 | 58.7081 |
| 14 | 5 | 4,696,401 | 0.0002 | 0.9990 | 57.7307 |
| 15 | 6 | 4,695,462 | 0.0002 | 0.9988 | 56.7528 |
| 16 | 7 | 4,694,523 | 0.0002 | 0.9986 | 55.8060 |
| 17 | 8 | 4,693,584 | 0.0002 | 0.9984 | 54.8584 |
| 18 | 9 | 4,692,646 | 0.0002 | 0.9982 | 53.9098 |
| 19 | 10 | 4,691,707 | 0.0002 | 0.9980 | 52.9603 |
