# Supplementary material for: Cost-effectiveness and budget impact analyses of dengue vaccination in Indonesia
Source: PLoS Negl Trop Dis. 2021 Aug 12;15(8):e0009664. doi: 10.1371/journal.pntd.0009664 (PMC8384188; doi:10.1371/journal.pntd.0009664)
Supplement: S2 Appendix — (PDF) [file pntd.0009664.s002.pdf]

UNDISCOUNTED

No Vaccination

age (year)	age (month)	DF						DHF						DSS						total
		primary infection			secondary infection			primary infection			secondary infection			primary infection			secondary infection			
		outpatient	hospitalization	death	outpatient	hospitalization	death	outpatient	hospitalization	death	outpatient	hospitalization	death	outpatient	hospitalization	death	outpatient	hospitalization	death	
**TOTAL**		**113,836**	**8,928**	**1,019**	**182,137**	**14,285**	**1,630**	**65,826**	**31,063**	**804**	**104,862**	**49,485**	**1,281**	**0**	**265**	**2**	**0**	**464**	**4**	**575,424**
9	-	10,359	812	93	16,574	1,300	148	5,990	2,827	73	9,542	4,503	117	-	24	0	-	42	0	52,364
10	1	10,357	812	93	16,571	1,300	148	5,989	2,826	73	9,541	4,502	117	-	24	0	-	42	0	52,353
11	2	10,355	812	93	16,568	1,299	148	5,988	2,826	73	9,539	4,501	117	-	24	0	-	42	0	52,343
12	3	10,353	812	93	16,565	1,299	148	5,987	2,825	73	9,537	4,500	117	-	24	0	-	42	0	52,332
13	4	10,351	812	93	16,561	1,299	148	5,985	2,825	73	9,535	4,499	116	-	24	0	-	42	0	52,322
14	5	10,349	812	93	16,558	1,299	148	5,984	2,824	73	9,533	4,499	116	-	24	0	-	42	0	52,311
15	6	10,347	812	93	16,555	1,298	148	5,983	2,823	73	9,531	4,498	116	-	24	0	-	42	0	52,301
16	7	10,345	811	93	16,551	1,298	148	5,982	2,823	73	9,529	4,497	116	-	24	0	-	42	0	52,290
17	8	10,342	811	93	16,548	1,298	148	5,981	2,822	73	9,527	4,496	116	-	24	0	-	42	0	52,280
18	9	10,340	811	93	16,545	1,298	148	5,979	2,822	73	9,525	4,495	116	-	24	0	-	42	0	52,269
19	10	10,338	811	93	16,541	1,297	148	5,978	2,821	73	9,523	4,494	116	-	24	0	-	42	0	52,259

Vaccination

age (year)	age (month)	DF						DHF						DSS						total
		primary infection			secondary infection			primary infection			secondary infection			primary infection			secondary infection			
		outpatient	hospitalization	death	outpatient	hospitalization	death	outpatient	hospitalization	death	outpatient	hospitalization	death	outpatient	hospitalization	death	outpatient	hospitalization	death	
**TOTAL**		**78,651**	**6,169**	**704**	**125,841**	**9,870**	**1,126**	**45,480**	**21,462**	**556**	**72,451**	**34,190**	**885**	**0**	**183**	**2**	**0**	**321**	**3**	**397,569**
9	-	7,157	561	64	11,452	898	103	4,139	1,953	51	6,593	3,111	81	0	17	0	0	29	0	36,179
10	1	7,156	561	64	11,449	898	102	4,138	1,953	51	6,592	3,111	81	0	17	0	0	29	0	36,172
11	2	7,154	561	64	11,447	898	102	4,137	1,952	51	6,590	3,110	81	0	17	0	0	29	0	36,164
12	3	7,153	561	64	11,445	898	102	4,136	1,952	51	6,589	3,109	80	0	17	0	0	29	0	36,157
13	4	7,152	561	64	11,442	897	102	4,135	1,951	51	6,588	3,109	80	0	17	0	0	29	0	36,150
14	5	7,150	561	64	11,440	897	102	4,135	1,951	51	6,586	3,108	80	0	17	0	0	29	0	36,143
15	6	7,149	561	64	11,438	897	102	4,134	1,951	51	6,585	3,108	80	0	17	0	0	29	0	36,135
16	7	7,147	561	64	11,436	897	102	4,133	1,950	50	6,584	3,107	80	0	17	0	0	29	0	36,128
17	8	7,146	560	64	11,433	897	102	4,132	1,950	50	6,582	3,106	80	0	17	0	0	29	0	36,121
18	9	7,144	560	64	11,431	897	102	4,131	1,950	50	6,581	3,106	80	0	17	0	0	29	0	36,114
19	10	7,143	560	64	11,429	896	102	4,130	1,949	50	6,580	3,105	80	0	17	0	0	29	0	36,107

DISCOUNTED

No Vaccination

age (year)	age (month)	DF						DHF						DSS						total
		primary infection			secondary infection			primary infection			secondary infection			primary infection			secondary infection			
		outpatient	hospitalization	death	outpatient	hospitalization	death	outpatient	hospitalization	death	outpatient	hospitalization	death	outpatient	hospitalization	death	outpatient	hospitalization	death	
**TOTAL**		**75,592**	**5,929**	**677**	**120,948**	**9,486**	**1,083**	**43,711**	**20,628**	**534**	**69,633**	**32,860**	**851**	**0**	**176**	**1**	**0**	**308**	**3**	**382,109**
9	-	7,939	623	71	12,703	996	114	4,591	2,166	56	7,313	3,451	89	0	18	0	0	32	0	40,132
10	1	7,707	604	69	12,331	967	110	4,456	2,103	54	7,099	3,350	87	0	18	0	0	31	0	38,956
11	2	7,481	587	67	11,969	939	107	4,326	2,041	53	6,891	3,252	84	0	17	0	0	30	0	37,813
12	3	7,261	570	65	11,618	911	104	4,199	1,981	51	6,689	3,156	82	0	17	0	0	30	0	36,705
13	4	7,048	553	63	11,277	885	101	4,076	1,923	50	6,493	3,064	79	0	16	0	0	29	0	35,629
14	5	6,842	537	61	10,947	859	98	3,956	1,867	48	6,302	2,974	77	0	16	0	0	28	0	34,584
15	6	6,641	521	59	10,626	833	95	3,840	1,812	47	6,118	2,887	75	0	15	0	0	27	0	33,570
16	7	6,446	506	58	10,314	809	92	3,728	1,759	46	5,938	2,802	73	0	15	0	0	26	0	32,586
17	8	6,257	491	56	10,012	785	90	3,618	1,708	44	5,764	2,720	70	0	15	0	0	26	0	31,630
18	9	6,074	476	54	9,718	762	87	3,512	1,657	43	5,595	2,640	68	0	14	0	0	25	0	30,703
19	10	5,896	462	53	9,433	740	84	3,409	1,609	42	5,431	2,563	66	0	14	0	0	24	0	29,803

Vaccination

age (year)	age (month)	DF						DHF						DSS						total
		primary infection			secondary infection			primary infection			secondary infection			primary infection			secondary infection			
		outpatient	hospitalization	death	outpatient	hospitalization	death	outpatient	hospitalization	death	outpatient	hospitalization	death	outpatient	hospitalization	death	outpatient	hospitalization	death	
**TOTAL**		**52,228**	**4,096**	**467**	**83,565**	**6,554**	**748**	**30,201**	**14,252**	**369**	**48,111**	**22,704**	**588**	**0**	**122**	**1**	**0**	**213**	**2**	**264,005**
9	-	5,485	430	49	8,777	688	79	3,172	1,497	39	5,053	2,385	62	0	13	0	0	22	0	27,728
10	1	5,325	418	48	8,519	668	76	3,079	1,453	38	4,905	2,315	60	0	12	0	0	22	0	26,915
11	2	5,168	405	46	8,270	649	74	2,989	1,410	37	4,761	2,247	58	0	12	0	0	21	0	26,126
12	3	5,017	393	45	8,027	630	72	2,901	1,369	35	4,621	2,181	56	0	12	0	0	20	0	25,360
13	4	4,870	382	44	7,792	611	70	2,816	1,329	34	4,486	2,117	55	0	11	0	0	20	0	24,616
14	5	4,727	371	42	7,563	593	68	2,733	1,290	33	4,354	2,055	53	0	11	0	0	19	0	23,895
15	6	4,588	360	41	7,342	576	66	2,653	1,252	32	4,227	1,995	52	0	11	0	0	19	0	23,194
16	7	4,454	349	40	7,126	559	64	2,575	1,215	31	4,103	1,936	50	0	10	0	0	18	0	22,514
17	8	4,323	339	39	6,917	543	62	2,500	1,180	31	3,983	1,879	49	0	10	0	0	18	0	21,854
18	9	4,197	329	38	6,714	527	60	2,427	1,145	30	3,866	1,824	47	0	10	0	0	17	0	21,213
19	10	4,074	319	36	6,518	511	58	2,356	1,112	29	3,752	1,771	46	0	9	0	0	17	0	20,591
