# Supplementary material for: Cost-effectiveness and budget impact analyses of dengue vaccination in Indonesia
Source: PLoS Negl Trop Dis. 2021 Aug 12;15(8):e0009664. doi: 10.1371/journal.pntd.0009664 (PMC8384188; doi:10.1371/journal.pntd.0009664)
Supplement: S3 Appendix — (PDF) [file pntd.0009664.s003.pdf]

UNDISCOUNTED

No Vaccination

age (year)	age (month)	DF						DHF						DSS						total
		primary infection			secondary infection			primary infection			secondary infection			primary infection			secondary infection			
		outpatient	hospitalization	death	outpatient	hospitalization	death	outpatient	hospitalization	death	outpatient	hospitalization	death	outpatient	hospitalization	death	outpatient	hospitalization	death	
**TOTAL**		**$2,188,049**	**$2,658,726**	**$0**	**$3,500,879**	**$4,253,962**	**$0**	**$1,265,242**	**$9,250,243**	**$0**	**$2,015,560**	**$14,735,853**	**$0**	**$0**	**$78,948**	**$0**	**$0**	**$138,159**	**$0**	**$40,085,623**
9	-	$199,113	$241,944	$0	$318,580	$387,111	$0	$115,137	$841,772	$0	$183,416	$1,340,963	$0	$0	$7,184	$0	$0	$12,572	$0	$3,647,793
10	1	$199,073	$241,896	$0	$318,516	$387,033	$0	$115,114	$841,604	$0	$183,379	$1,340,695	$0	$0	$7,183	$0	$0	$12,570	$0	$3,647,063
11	2	$199,033	$241,847	$0	$318,453	$386,956	$0	$115,091	$841,436	$0	$183,343	$1,340,427	$0	$0	$7,181	$0	$0	$12,567	$0	$3,646,334
12	3	$198,993	$241,799	$0	$318,389	$386,878	$0	$115,068	$841,267	$0	$183,306	$1,340,159	$0	$0	$7,180	$0	$0	$12,565	$0	$3,645,605
13	4	$198,953	$241,751	$0	$318,325	$386,801	$0	$115,045	$841,099	$0	$183,269	$1,339,891	$0	$0	$7,179	$0	$0	$12,562	$0	$3,644,876
14	5	$198,914	$241,702	$0	$318,262	$386,724	$0	$115,022	$840,931	$0	$183,233	$1,339,623	$0	$0	$7,177	$0	$0	$12,560	$0	$3,644,147
15	6	$198,874	$241,654	$0	$318,198	$386,646	$0	$114,999	$840,763	$0	$183,196	$1,339,355	$0	$0	$7,176	$0	$0	$12,557	$0	$3,643,418
16	7	$198,834	$241,606	$0	$318,134	$386,569	$0	$114,976	$840,595	$0	$183,159	$1,339,087	$0	$0	$7,174	$0	$0	$12,555	$0	$3,642,689
17	8	$198,794	$241,557	$0	$318,071	$386,492	$0	$114,953	$840,427	$0	$183,123	$1,338,819	$0	$0	$7,173	$0	$0	$12,552	$0	$3,641,961
18	9	$198,754	$241,509	$0	$318,007	$386,415	$0	$114,930	$840,259	$0	$183,086	$1,338,551	$0	$0	$7,171	$0	$0	$12,550	$0	$3,641,233
19	10	$198,715	$241,461	$0	$317,944	$386,337	$0	$114,907	$840,091	$0	$183,050	$1,338,284	$0	$0	$7,170	$0	$0	$12,547	$0	$3,640,504

Vaccination

age (year)	age (month)	DF						DHF						DSS						total
		primary infection			secondary infection			primary infection			secondary infection			primary infection			secondary infection			
		outpatient	hospitalization	death	outpatient	hospitalization	death	outpatient	hospitalization	death	outpatient	hospitalization	death	outpatient	hospitalization	death	outpatient	hospitalization	death	
**TOTAL**		**$1,511,758**	**$1,836,956**	**$0**	**$2,418,812**	**$2,939,130**	**$0**	**$874,176**	**$6,391,139**	**$0**	**$1,392,583**	**$10,181,233**	**$0**	**$0**	**$54,546**	**$0**	**$0**	**$95,456**	**$0**	**$27,695,790**
9	-	$137,570	$167,163	$0	$220,112	$267,461	$0	$79,550	$581,594	$0	$126,725	$926,493	$0	$0	$4,964	$0	$0	$8,687	$0	$2,520,318
10	1	$137,542	$167,130	$0	$220,068	$267,407	$0	$79,534	$581,478	$0	$126,700	$926,307	$0	$0	$4,963	$0	$0	$8,685	$0	$2,519,814
11	2	$137,515	$167,096	$0	$220,024	$267,354	$0	$79,518	$581,361	$0	$126,674	$926,122	$0	$0	$4,962	$0	$0	$8,683	$0	$2,519,310
12	3	$137,487	$167,063	$0	$219,980	$267,300	$0	$79,502	$581,245	$0	$126,649	$925,937	$0	$0	$4,961	$0	$0	$8,681	$0	$2,518,806
13	4	$137,460	$167,029	$0	$219,936	$267,247	$0	$79,486	$581,129	$0	$126,624	$925,752	$0	$0	$4,960	$0	$0	$8,680	$0	$2,518,302
14	5	$137,432	$166,996	$0	$219,892	$267,194	$0	$79,471	$581,013	$0	$126,598	$925,566	$0	$0	$4,959	$0	$0	$8,678	$0	$2,517,799
15	6	$137,405	$166,963	$0	$219,848	$267,140	$0	$79,455	$580,896	$0	$126,573	$925,381	$0	$0	$4,958	$0	$0	$8,676	$0	$2,517,295
16	7	$137,378	$166,929	$0	$219,804	$267,087	$0	$79,439	$580,780	$0	$126,548	$925,196	$0	$0	$4,957	$0	$0	$8,674	$0	$2,516,792
17	8	$137,350	$166,896	$0	$219,760	$267,033	$0	$79,423	$580,664	$0	$126,522	$925,011	$0	$0	$4,956	$0	$0	$8,673	$0	$2,516,288
18	9	$137,323	$166,862	$0	$219,716	$266,980	$0	$79,407	$580,548	$0	$126,497	$924,826	$0	$0	$4,955	$0	$0	$8,671	$0	$2,515,785
19	10	$137,295	$166,829	$0	$219,672	$266,926	$0	$79,391	$580,432	$0	$126,472	$924,641	$0	$0	$4,954	$0	$0	$8,669	$0	$2,515,282

DISCOUNTED

No Vaccination

age (year)	age (month)	DF						DHF						DSS						total
		primary infection			secondary infection			primary infection			secondary infection			primary infection			secondary infection			
		outpatient	hospitalization	death	outpatient	hospitalization	death	outpatient	hospitalization	death	outpatient	hospitalization	death	outpatient	hospitalization	death	outpatient	hospitalization	death	
**TOTAL**		**$1,452,972**	**$1,765,524**	**$0**	**$2,324,755**	**$2,824,839**	**$0**	**$840,183**	**$6,142,614**	**$0**	**$1,338,431**	**$9,785,327**	**$0**	**$0**	**$52,425**	**$0**	**$0**	**$91,744**	**$0**	**$26,618,814**
9	-	$152,603	$185,430	$0	$244,165	$296,688	$0	$88,243	$645,148	$0	$140,573	$1,027,736	$0	$0	$5,506	$0	$0	$9,636	$0	$2,795,729
10	1	$148,129	$179,993	$0	$237,006	$287,989	$0	$85,656	$626,232	$0	$136,451	$997,603	$0	$0	$5,345	$0	$0	$9,353	$0	$2,713,758
11	2	$143,786	$174,716	$0	$230,057	$279,545	$0	$83,144	$607,871	$0	$132,451	$968,353	$0	$0	$5,188	$0	$0	$9,079	$0	$2,634,189
12	3	$139,570	$169,593	$0	$223,312	$271,349	$0	$80,706	$590,048	$0	$128,567	$939,960	$0	$0	$5,036	$0	$0	$8,813	$0	$2,556,954
13	4	$135,478	$164,620	$0	$216,764	$263,393	$0	$78,340	$572,748	$0	$124,798	$912,400	$0	$0	$4,888	$0	$0	$8,554	$0	$2,481,983
14	5	$131,505	$159,794	$0	$210,408	$255,670	$0	$76,043	$555,954	$0	$121,138	$885,648	$0	$0	$4,745	$0	$0	$8,304	$0	$2,409,210
15	6	$127,649	$155,109	$0	$204,239	$248,174	$0	$73,813	$539,654	$0	$117,587	$859,681	$0	$0	$4,606	$0	$0	$8,060	$0	$2,338,571
16	7	$123,907	$150,561	$0	$198,251	$240,897	$0	$71,649	$523,831	$0	$114,139	$834,475	$0	$0	$4,471	$0	$0	$7,824	$0	$2,270,004
17	8	$120,274	$146,146	$0	$192,438	$233,834	$0	$69,548	$508,472	$0	$110,792	$810,008	$0	$0	$4,340	$0	$0	$7,594	$0	$2,203,446
18	9	$116,747	$141,861	$0	$186,796	$226,978	$0	$67,509	$493,563	$0	$107,544	$786,258	$0	$0	$4,212	$0	$0	$7,372	$0	$2,138,840
19	10	$113,324	$137,702	$0	$181,319	$220,323	$0	$65,530	$479,092	$0	$104,391	$763,205	$0	$0	$4,089	$0	$0	$7,156	$0	$2,076,129

Vaccination

age (year)	age (month)	DF						DHF						DSS						total
		primary infection			secondary infection			primary infection			secondary infection			primary infection			secondary infection			
		outpatient	hospitalization	death	outpatient	hospitalization	death	outpatient	hospitalization	death	outpatient	hospitalization	death	outpatient	hospitalization	death	outpatient	hospitalization	death	
**TOTAL**		**$1,003,881**	**$1,219,829**	**$0**	**$1,606,210**	**$1,951,726**	**$0**	**$580,496**	**$4,244,029**	**$0**	**$924,743**	**$6,760,837**	**$0**	**$0**	**$36,221**	**$0**	**$0**	**$63,388**	**$0**	**$18,391,359**
9	-	$105,436	$128,117	$0	$168,698	$204,986	$0	$60,968	$445,743	$0	$97,124	$710,079	$0	$0	$3,804	$0	$0	$6,657	$0	$1,931,614
10	1	$102,345	$124,360	$0	$163,751	$198,976	$0	$59,181	$432,674	$0	$94,276	$689,260	$0	$0	$3,693	$0	$0	$6,462	$0	$1,874,978
11	2	$99,344	$120,714	$0	$158,950	$193,142	$0	$57,446	$419,988	$0	$91,512	$669,050	$0	$0	$3,584	$0	$0	$6,273	$0	$1,820,003
12	3	$96,431	$117,174	$0	$154,290	$187,479	$0	$55,761	$407,674	$0	$88,829	$649,433	$0	$0	$3,479	$0	$0	$6,089	$0	$1,766,640
13	4	$93,604	$113,739	$0	$149,766	$181,982	$0	$54,126	$395,720	$0	$86,225	$630,392	$0	$0	$3,377	$0	$0	$5,910	$0	$1,714,841
14	5	$90,859	$110,404	$0	$145,375	$176,646	$0	$52,539	$384,118	$0	$83,696	$611,908	$0	$0	$3,278	$0	$0	$5,737	$0	$1,664,561
15	6	$88,195	$107,167	$0	$141,112	$171,467	$0	$50,999	$372,855	$0	$81,242	$593,967	$0	$0	$3,182	$0	$0	$5,569	$0	$1,615,756
16	7	$85,609	$104,025	$0	$136,975	$166,440	$0	$49,504	$361,923	$0	$78,860	$576,552	$0	$0	$3,089	$0	$0	$5,406	$0	$1,568,381
17	8	$83,099	$100,975	$0	$132,958	$161,560	$0	$48,052	$351,311	$0	$76,548	$559,647	$0	$0	$2,998	$0	$0	$5,247	$0	$1,522,396
18	9	$80,663	$98,014	$0	$129,060	$156,823	$0	$46,643	$341,011	$0	$74,304	$543,238	$0	$0	$2,910	$0	$0	$5,093	$0	$1,477,759
19	10	$78,297	$95,140	$0	$125,276	$152,224	$0	$45,276	$331,012	$0	$72,125	$527,310	$0	$0	$2,825	$0	$0	$4,944	$0	$1,434,430
