# Supplementary material for: Cost-effectiveness and budget impact analyses of dengue vaccination in Indonesia
Source: PLoS Negl Trop Dis. 2021 Aug 12;15(8):e0009664. doi: 10.1371/journal.pntd.0009664 (PMC8384188; doi:10.1371/journal.pntd.0009664)
Supplement: S4 Appendix — (PDF) [file pntd.0009664.s004.pdf]

UNDISCOUNTED

No Vaccination

age (year)	age (month)	DF						DHF						DSS						total
		primary infection			secondary infection			primary infection			secondary infection			primary infection			secondary infection			
		outpatient	hospitalization	death	outpatient	hospitalization	death	outpatient	hospitalization	death	outpatient	hospitalization	death	outpatient	hospitalization	death	outpatient	hospitalization	death	
**TOTAL**		**$70,053**	**$2,035,147**	**$0**	**$112,084**	**$3,256,236**	**$0**	**$40,508**	**$7,080,686**	**$0**	**$64,530**	**$11,279,698**	**$0**	**$0**	**$60,432**	**$0**	**$0**	**$105,755**	**$0**	**$24,105,129**
9	-	$6,375	$185,198	$0	$10,200	$296,318	$0	$3,686	$644,343	$0	$5,872	$1,026,453	$0	$0	$5,499	$0	$0	$9,624	$0	$2,193,567
10	1	$6,374	$185,161	$0	$10,198	$296,258	$0	$3,686	$644,214	$0	$5,871	$1,026,248	$0	$0	$5,498	$0	$0	$9,622	$0	$2,193,129
11	2	$6,372	$185,124	$0	$10,196	$296,199	$0	$3,685	$644,085	$0	$5,870	$1,026,042	$0	$0	$5,497	$0	$0	$9,620	$0	$2,192,690
12	3	$6,371	$185,087	$0	$10,194	$296,140	$0	$3,684	$643,956	$0	$5,869	$1,025,837	$0	$0	$5,496	$0	$0	$9,618	$0	$2,192,252
13	4	$6,370	$185,050	$0	$10,192	$296,081	$0	$3,683	$643,827	$0	$5,868	$1,025,632	$0	$0	$5,495	$0	$0	$9,616	$0	$2,191,813
14	5	$6,368	$185,013	$0	$10,189	$296,021	$0	$3,683	$643,699	$0	$5,866	$1,025,427	$0	$0	$5,494	$0	$0	$9,614	$0	$2,191,375
15	6	$6,367	$184,976	$0	$10,187	$295,962	$0	$3,682	$643,570	$0	$5,865	$1,025,222	$0	$0	$5,493	$0	$0	$9,612	$0	$2,190,937
16	7	$6,366	$184,939	$0	$10,185	$295,903	$0	$3,681	$643,441	$0	$5,864	$1,025,017	$0	$0	$5,492	$0	$0	$9,610	$0	$2,190,499
17	8	$6,365	$184,902	$0	$10,183	$295,844	$0	$3,680	$643,313	$0	$5,863	$1,024,812	$0	$0	$5,490	$0	$0	$9,608	$0	$2,190,060
18	9	$6,363	$184,865	$0	$10,181	$295,785	$0	$3,680	$643,184	$0	$5,862	$1,024,607	$0	$0	$5,489	$0	$0	$9,606	$0	$2,189,623
19	10	$6,362	$184,828	$0	$10,179	$295,725	$0	$3,679	$643,055	$0	$5,861	$1,024,402	$0	$0	$5,488	$0	$0	$9,604	$0	$2,189,185

Vaccination

age (year)	age (month)	DF						DHF						DSS						total
		primary infection			secondary infection			primary infection			secondary infection			primary infection			secondary infection			
		outpatient	hospitalization	death	outpatient	hospitalization	death	outpatient	hospitalization	death	outpatient	hospitalization	death	outpatient	hospitalization	death	outpatient	hospitalization	death	
**TOTAL**		**$48,401**	**$1,406,115**	**$0**	**$77,441**	**$2,249,785**	**$0**	**$27,988**	**$4,892,158**	**$0**	**$44,585**	**$7,793,321**	**$0**	**$0**	**$41,753**	**$0**	**$0**	**$73,068**	**$0**	**$16,654,614**
9	-	$4,404	$127,957	$0	$7,047	$204,730	$0	$2,547	$445,186	$0	$4,057	$709,192	$0	$0	$3,800	$0	$0	$6,649	$0	$1,515,570
10	1	$4,404	$127,931	$0	$7,046	$204,690	$0	$2,546	$445,097	$0	$4,056	$709,051	$0	$0	$3,799	$0	$0	$6,648	$0	$1,515,267
11	2	$4,403	$127,905	$0	$7,044	$204,649	$0	$2,546	$445,008	$0	$4,056	$708,909	$0	$0	$3,798	$0	$0	$6,647	$0	$1,514,964
12	3	$4,402	$127,880	$0	$7,043	$204,608	$0	$2,545	$444,919	$0	$4,055	$708,767	$0	$0	$3,797	$0	$0	$6,645	$0	$1,514,661
13	4	$4,401	$127,854	$0	$7,041	$204,567	$0	$2,545	$444,830	$0	$4,054	$708,625	$0	$0	$3,796	$0	$0	$6,644	$0	$1,514,358
14	5	$4,400	$127,829	$0	$7,040	$204,526	$0	$2,544	$444,742	$0	$4,053	$708,484	$0	$0	$3,796	$0	$0	$6,643	$0	$1,514,056
15	6	$4,399	$127,803	$0	$7,039	$204,485	$0	$2,544	$444,653	$0	$4,052	$708,342	$0	$0	$3,795	$0	$0	$6,641	$0	$1,513,753
16	7	$4,398	$127,778	$0	$7,037	$204,444	$0	$2,543	$444,564	$0	$4,052	$708,200	$0	$0	$3,794	$0	$0	$6,640	$0	$1,513,450
17	8	$4,397	$127,752	$0	$7,036	$204,403	$0	$2,543	$444,475	$0	$4,051	$708,059	$0	$0	$3,793	$0	$0	$6,639	$0	$1,513,147
18	9	$4,397	$127,726	$0	$7,034	$204,362	$0	$2,542	$444,386	$0	$4,050	$707,917	$0	$0	$3,793	$0	$0	$6,637	$0	$1,512,845
19	10	$4,396	$127,701	$0	$7,033	$204,321	$0	$2,542	$444,297	$0	$4,049	$707,775	$0	$0	$3,792	$0	$0	$6,636	$0	$1,512,542

DISCOUNTED

No Vaccination

age (year)	age (month)	DF						DHF						DSS						total
		primary infection			secondary infection			primary infection			secondary infection			primary infection			secondary infection			
		outpatient	hospitalization	death	outpatient	hospitalization	death	outpatient	hospitalization	death	outpatient	hospitalization	death	outpatient	hospitalization	death	outpatient	hospitalization	death	
**TOTAL**		**$46,518**	**$1,351,437**	**$0**	**$74,430**	**$2,162,300**	**$0**	**$26,899**	**$4,701,922**	**$0**	**$42,851**	**$7,490,271**	**$0**	**$0**	**$40,129**	**$0**	**$0**	**$70,227**	**$0**	**$16,006,984**
9	-	$4,886	$141,939	$0	$7,817	$227,103	$0	$2,825	$493,835	$0	$4,501	$786,691	$0	$0	$4,215	$0	$0	$7,376	$0	$1,681,187
10	1	$4,743	$137,777	$0	$7,588	$220,444	$0	$2,742	$479,356	$0	$4,369	$763,625	$0	$0	$4,091	$0	$0	$7,160	$0	$1,631,894
11	2	$4,603	$133,738	$0	$7,366	$213,980	$0	$2,662	$465,301	$0	$4,241	$741,235	$0	$0	$3,971	$0	$0	$6,950	$0	$1,584,046
12	3	$4,468	$129,817	$0	$7,150	$207,706	$0	$2,584	$451,658	$0	$4,116	$719,502	$0	$0	$3,855	$0	$0	$6,746	$0	$1,537,601
13	4	$4,337	$126,010	$0	$6,940	$201,616	$0	$2,508	$438,415	$0	$3,996	$698,405	$0	$0	$3,742	$0	$0	$6,548	$0	$1,492,518
14	5	$4,210	$122,316	$0	$6,736	$195,705	$0	$2,435	$425,561	$0	$3,878	$677,928	$0	$0	$3,632	$0	$0	$6,356	$0	$1,448,757
15	6	$4,087	$118,729	$0	$6,539	$189,967	$0	$2,363	$413,083	$0	$3,765	$658,051	$0	$0	$3,526	$0	$0	$6,170	$0	$1,406,279
16	7	$3,967	$115,248	$0	$6,347	$184,397	$0	$2,294	$400,971	$0	$3,654	$638,757	$0	$0	$3,422	$0	$0	$5,989	$0	$1,365,046
17	8	$3,851	$111,869	$0	$6,161	$178,990	$0	$2,227	$389,215	$0	$3,547	$620,028	$0	$0	$3,322	$0	$0	$5,813	$0	$1,325,023
18	9	$3,738	$108,589	$0	$5,980	$173,742	$0	$2,161	$377,803	$0	$3,443	$601,849	$0	$0	$3,224	$0	$0	$5,643	$0	$1,286,172
19	10	$3,628	$105,405	$0	$5,805	$168,648	$0	$2,098	$366,725	$0	$3,342	$584,202	$0	$0	$3,130	$0	$0	$5,477	$0	$1,248,461

Vaccination

age (year)	age (month)	DF						DHF						DSS						total
		primary infection			secondary infection			primary infection			secondary infection			primary infection			secondary infection			
		outpatient	hospitalization	death	outpatient	hospitalization	death	outpatient	hospitalization	death	outpatient	hospitalization	death	outpatient	hospitalization	death	outpatient	hospitalization	death	
**TOTAL**		**$32,140**	**$933,729**	**$0**	**$51,425**	**$1,493,967**	**$0**	**$18,585**	**$3,248,632**	**$0**	**$29,607**	**$5,175,146**	**$0**	**$0**	**$27,726**	**$0**	**$0**	**$48,521**	**$0**	**$11,059,478**
9	-	$3,376	$98,068	$0	$5,401	$156,909	$0	$1,952	$341,198	$0	$3,110	$543,537	$0	$0	$2,912	$0	$0	$5,096	$0	$1,161,558
10	1	$3,277	$95,193	$0	$5,243	$152,308	$0	$1,895	$331,194	$0	$3,018	$527,600	$0	$0	$2,827	$0	$0	$4,947	$0	$1,127,501
11	2	$3,181	$92,402	$0	$5,089	$147,842	$0	$1,839	$321,484	$0	$2,930	$512,131	$0	$0	$2,744	$0	$0	$4,802	$0	$1,094,442
12	3	$3,087	$89,692	$0	$4,940	$143,508	$0	$1,785	$312,058	$0	$2,844	$497,115	$0	$0	$2,663	$0	$0	$4,661	$0	$1,062,353
13	4	$2,997	$87,062	$0	$4,795	$139,300	$0	$1,733	$302,908	$0	$2,761	$482,539	$0	$0	$2,585	$0	$0	$4,524	$0	$1,031,204
14	5	$2,909	$84,510	$0	$4,654	$135,216	$0	$1,682	$294,027	$0	$2,680	$468,391	$0	$0	$2,509	$0	$0	$4,391	$0	$1,000,969
15	6	$2,824	$82,032	$0	$4,518	$131,251	$0	$1,633	$285,406	$0	$2,601	$454,658	$0	$0	$2,436	$0	$0	$4,263	$0	$971,620
16	7	$2,741	$79,627	$0	$4,385	$127,403	$0	$1,585	$277,037	$0	$2,525	$441,327	$0	$0	$2,364	$0	$0	$4,138	$0	$943,132
17	8	$2,661	$77,292	$0	$4,257	$123,667	$0	$1,538	$268,915	$0	$2,451	$428,387	$0	$0	$2,295	$0	$0	$4,016	$0	$915,479
18	9	$2,582	$75,026	$0	$4,132	$120,041	$0	$1,493	$261,030	$0	$2,379	$415,827	$0	$0	$2,228	$0	$0	$3,899	$0	$888,637
19	10	$2,507	$72,826	$0	$4,011	$116,522	$0	$1,450	$253,376	$0	$2,309	$403,634	$0	$0	$2,162	$0	$0	$3,784	$0	$862,582
