# Supplementary material for: Cost-effectiveness and budget impact analyses of dengue vaccination in Indonesia
Source: PLoS Negl Trop Dis. 2021 Aug 12;15(8):e0009664. doi: 10.1371/journal.pntd.0009664 (PMC8384188; doi:10.1371/journal.pntd.0009664)
Supplement: S5 Appendix — (PDF) [file pntd.0009664.s005.pdf]

age (year)	age (month)	Vaccinate?	Vaccination Cost	
			**UNDISCOUNTED**	**DISCOUNTED**
		**TOTAL**	**$441,180,274**	**$334,437,563**
9	-	1.00	$147,074,797	$112,720,586
9	0.5	1.00	$147,060,091	$112,570,585
10	1	1.00	$147,045,385	$109,146,392
11	2	N	$0	$0
12	3	N	$0	$0
13	4	N	$0	$0
14	5	N	$0	$0
15	6	N	$0	$0
16	7	N	$0	$0
17	8	N	$0	$0
18	9	N	$0	$0
19	10	N	$0	$0

cohort size * vaccine coverage * cost/dose
