# Supplementary material for: Cost-effectiveness and budget impact analyses of dengue vaccination in Indonesia
Source: PLoS Negl Trop Dis. 2021 Aug 12;15(8):e0009664. doi: 10.1371/journal.pntd.0009664 (PMC8384188; doi:10.1371/journal.pntd.0009664)
Supplement: S6 Appendix — (PDF) [file pntd.0009664.s006.pdf]

UNDISCOUNTED

No Vaccination

age (year)	age (month)	DF						DHF						DSS						total non-fatal	total fatal
		primary infection			secondary infection			primary infection			secondary infection			primary infection			secondary infection				
		outpatient	hospitalization	death	outpatient	hospitalization	death	outpatient	hospitalization	death	outpatient	hospitalization	death	outpatient	hospitalization	death	outpatient	hospitalization	death		
**TOTAL**		**4.76**	**1.64**	**58,852.83**	**7.62**	**2.62**	**95,522.81**	**2.75**	**5.70**	**46,448.39**	**4.39**	**9.09**	**75,060.68**	**0.00**	**0.05**	**0.00**	**0.00**	**0.09**	**0.00**	**38.72**	**275,884.71**
9	-	0.43	0.15	5,805.81	0.69	0.24	9,289.29	0.25	0.52	4,582.11	0.40	0.83	7,299.41	0.00	0.00	0.00	0.00	0.01	0.00	3.52	26,976.62
10	1	0.43	0.15	5,714.18	0.69	0.24	9,215.06	0.25	0.52	4,509.80	0.40	0.83	7,241.09	0.00	0.00	0.00	0.00	0.01	0.00	3.52	26,680.14
11	2	0.43	0.15	5,622.55	0.69	0.24	9,140.87	0.25	0.52	4,437.48	0.40	0.83	7,182.78	0.00	0.00	0.00	0.00	0.01	0.00	3.52	26,383.68
12	3	0.43	0.15	5,530.91	0.69	0.24	8,994.28	0.25	0.52	4,365.16	0.40	0.83	7,067.60	0.00	0.00	0.00	0.00	0.01	0.00	3.52	25,957.95
13	4	0.43	0.15	5,439.28	0.69	0.24	8,847.69	0.25	0.52	4,292.84	0.40	0.83	6,952.41	0.00	0.00	0.00	0.00	0.01	0.00	3.52	25,532.23
14	5	0.43	0.15	5,347.65	0.69	0.24	8,701.11	0.25	0.52	4,220.52	0.40	0.83	6,837.23	0.00	0.00	0.00	0.00	0.01	0.00	3.52	25,106.51
15	6	0.43	0.15	5,256.02	0.69	0.24	8,554.53	0.25	0.52	4,148.20	0.40	0.83	6,722.05	0.00	0.00	0.00	0.00	0.01	0.00	3.52	24,680.80
16	7	0.43	0.15	5,167.30	0.69	0.24	8,407.95	0.25	0.52	4,078.19	0.40	0.83	6,606.87	0.00	0.00	0.00	0.00	0.01	0.00	3.52	24,260.30
17	8	0.43	0.15	5,078.54	0.69	0.24	8,266.03	0.25	0.52	4,008.13	0.40	0.83	6,495.35	0.00	0.00	0.00	0.00	0.01	0.00	3.52	23,848.05
18	9	0.43	0.15	4,989.72	0.69	0.24	8,124.04	0.25	0.52	3,938.04	0.40	0.83	6,383.77	0.00	0.00	0.00	0.00	0.01	0.00	3.52	23,435.57
19	10	0.43	0.15	4,900.86	0.69	0.24	7,981.96	0.25	0.52	3,867.91	0.40	0.83	6,272.13	0.00	0.00	0.00	0.00	0.01	0.00	3.52	23,022.86

Vaccination

age (year)	age (month)	DF						DHF						DSS						total non-fatal	total fatal
		primary infection			secondary infection			primary infection			secondary infection			primary infection			secondary infection				
		outpatient	hospitalization	death	outpatient	hospitalization	death	outpatient	hospitalization	death	outpatient	hospitalization	death	outpatient	hospitalization	death	outpatient	hospitalization	death		
**TOTAL**		**3.29**	**1.13**	**40,662.35**	**5.27**	**1.81**	**65,998.22**	**1.90**	**3.94**	**32,091.93**	**3.03**	**6.28**	**51,860.61**	**0.00**	**0.03**	**0.00**	**0.00**	**0.06**	**0.00**	**26.75**	**190,613.10**
9	-	0.30	0.10	4,011.32	0.48	0.16	6,418.12	0.17	0.36	3,165.85	0.28	0.57	5,043.28	0.00	0.00	0.00	0.00	0.01	0.00	2.43	18,638.57
10	1	0.30	0.10	3,948.02	0.48	0.16	6,366.83	0.17	0.36	3,115.89	0.28	0.57	5,002.98	0.00	0.00	0.00	0.00	0.01	0.00	2.43	18,433.73
11	2	0.30	0.10	3,884.71	0.48	0.16	6,315.57	0.17	0.36	3,065.93	0.28	0.57	4,962.70	0.00	0.00	0.00	0.00	0.01	0.00	2.43	18,228.90
12	3	0.30	0.10	3,821.40	0.48	0.16	6,214.29	0.17	0.36	3,015.96	0.28	0.57	4,883.11	0.00	0.00	0.00	0.00	0.01	0.00	2.43	17,934.76
13	4	0.30	0.10	3,758.09	0.48	0.16	6,113.01	0.17	0.36	2,965.99	0.28	0.57	4,803.53	0.00	0.00	0.00	0.00	0.01	0.00	2.43	17,640.62
14	5	0.30	0.10	3,694.78	0.48	0.16	6,011.73	0.17	0.36	2,916.03	0.28	0.57	4,723.95	0.00	0.00	0.00	0.00	0.01	0.00	2.43	17,346.48
15	6	0.30	0.10	3,631.47	0.48	0.16	5,910.46	0.17	0.36	2,866.06	0.28	0.57	4,644.37	0.00	0.00	0.00	0.00	0.01	0.00	2.43	17,052.35
16	7	0.30	0.10	3,570.17	0.48	0.16	5,809.18	0.17	0.36	2,817.68	0.28	0.57	4,564.79	0.00	0.00	0.00	0.00	0.01	0.00	2.43	16,761.83
17	8	0.30	0.10	3,508.84	0.48	0.16	5,711.13	0.17	0.36	2,769.28	0.28	0.57	4,487.74	0.00	0.00	0.00	0.00	0.01	0.00	2.43	16,476.99
18	9	0.30	0.10	3,447.48	0.48	0.16	5,613.02	0.17	0.36	2,720.85	0.28	0.57	4,410.65	0.00	0.00	0.00	0.00	0.01	0.00	2.43	16,192.00
19	10	0.30	0.10	3,386.08	0.48	0.16	5,514.86	0.17	0.36	2,672.40	0.28	0.57	4,333.51	0.00	0.00	0.00	0.00	0.01	0.00	2.43	15,906.86

DISCOUNTED

No Vaccination

age (year)	age (month)	DF						DHF						DSS						total non-fatal	total fatal
		primary infection			secondary infection			primary infection			secondary infection			primary infection			secondary infection				
		outpatient	hospitalization	death	outpatient	hospitalization	death	outpatient	hospitalization	death	outpatient	hospitalization	death	outpatient	hospitalization	death	outpatient	hospitalization	death		
**TOTAL**		**3.16**	**1.09**	**39,274.15**	**5.06**	**1.74**	**63,721.09**	**1.83**	**3.79**	**30,996.32**	**2.91**	**6.03**	**50,071.27**	**0.00**	**0.03**	**0.00**	**0.00**	**0.06**	**0.00**	**25.71**	**184,062.83**
9	-	0.33	0.11	4,449.67	0.53	0.18	7,119.47	0.19	0.40	3,511.81	0.31	0.63	5,594.39	0.00	0.00	0.00	0.00	0.01	0.00	2.70	20,675.33
10	1	0.32	0.11	4,251.89	0.52	0.18	6,856.87	0.19	0.39	3,355.72	0.30	0.62	5,388.05	0.00	0.00	0.00	0.00	0.01	0.00	2.62	19,852.53
11	2	0.31	0.11	4,061.85	0.50	0.17	6,603.56	0.18	0.37	3,205.73	0.29	0.60	5,189.00	0.00	0.00	0.00	0.00	0.01	0.00	2.54	19,060.13
12	3	0.30	0.10	3,879.27	0.49	0.17	6,308.41	0.18	0.36	3,061.64	0.28	0.58	4,957.07	0.00	0.00	0.00	0.00	0.01	0.00	2.47	18,206.38
13	4	0.29	0.10	3,703.89	0.47	0.16	6,024.85	0.17	0.35	2,923.22	0.27	0.56	4,734.25	0.00	0.00	0.00	0.00	0.01	0.00	2.40	17,386.21
14	5	0.29	0.10	3,535.43	0.46	0.16	5,752.46	0.17	0.34	2,790.26	0.26	0.55	4,520.21	0.00	0.00	0.00	0.00	0.01	0.00	2.33	16,598.36
15	6	0.28	0.10	3,373.64	0.44	0.15	5,490.83	0.16	0.33	2,662.57	0.26	0.53	4,314.63	0.00	0.00	0.00	0.00	0.00	0.00	2.26	15,841.66
16	7	0.27	0.09	3,220.09	0.43	0.15	5,239.56	0.16	0.32	2,541.39	0.25	0.51	4,117.18	0.00	0.00	0.00	0.00	0.00	0.00	2.19	15,118.22
17	8	0.26	0.09	3,072.60	0.42	0.14	5,001.08	0.15	0.31	2,424.99	0.24	0.50	3,929.79	0.00	0.00	0.00	0.00	0.00	0.00	2.13	14,428.46
18	9	0.25	0.09	2,930.94	0.41	0.14	4,772.01	0.15	0.30	2,313.18	0.23	0.48	3,749.79	0.00	0.00	0.00	0.00	0.00	0.00	2.07	13,765.93
19	10	0.25	0.08	2,794.89	0.39	0.14	4,552.00	0.14	0.30	2,205.81	0.23	0.47	3,576.91	0.00	0.00	0.00	0.00	0.00	0.00	2.01	13,129.62

Vaccination

age (year)	age (month)	DF						DHF						DSS						total non-fatal	total fatal
		primary infection			secondary infection			primary infection			secondary infection			primary infection			secondary infection				
		outpatient	hospitalization	death	outpatient	hospitalization	death	outpatient	hospitalization	death	outpatient	hospitalization	death	outpatient	hospitalization	death	outpatient	hospitalization	death		
**TOTAL**		**2.19**	**0.75**	**27,135.13**	**3.50**	**1.20**	**44,025.91**	**1.26**	**2.62**	**21,415.85**	**2.01**	**4.17**	**34,595.03**	**0.00**	**0.02**	**0.00**	**0.00**	**0.04**	**0.00**	**17.76**	**127,171.92**
9	-	0.23	0.08	3,074.35	0.37	0.13	4,918.95	0.13	0.27	2,426.36	0.21	0.44	3,865.25	0.00	0.00	0.00	0.00	0.00	0.00	1.87	14,284.91
10	1	0.22	0.08	2,937.70	0.36	0.12	4,737.52	0.13	0.27	2,318.52	0.21	0.43	3,722.69	0.00	0.00	0.00	0.00	0.00	0.00	1.81	13,716.42
11	2	0.22	0.07	2,806.40	0.35	0.12	4,562.50	0.13	0.26	2,214.89	0.20	0.41	3,585.16	0.00	0.00	0.00	0.00	0.00	0.00	1.76	13,168.95
12	3	0.21	0.07	2,680.25	0.34	0.12	4,358.58	0.12	0.25	2,115.33	0.19	0.40	3,424.92	0.00	0.00	0.00	0.00	0.00	0.00	1.71	12,579.08
13	4	0.20	0.07	2,559.07	0.33	0.11	4,162.66	0.12	0.24	2,019.70	0.19	0.39	3,270.97	0.00	0.00	0.00	0.00	0.00	0.00	1.66	12,012.40
14	5	0.20	0.07	2,442.68	0.32	0.11	3,974.46	0.11	0.24	1,927.84	0.18	0.38	3,123.09	0.00	0.00	0.00	0.00	0.00	0.00	1.61	11,468.07
15	6	0.19	0.07	2,330.90	0.31	0.11	3,793.70	0.11	0.23	1,839.62	0.18	0.37	2,981.04	0.00	0.00	0.00	0.00	0.00	0.00	1.56	10,945.26
16	7	0.19	0.06	2,224.81	0.30	0.10	3,620.09	0.11	0.22	1,755.89	0.17	0.36	2,844.62	0.00	0.00	0.00	0.00	0.00	0.00	1.51	10,445.42
17	8	0.18	0.06	2,122.91	0.29	0.10	3,455.33	0.10	0.22	1,675.46	0.17	0.35	2,715.16	0.00	0.00	0.00	0.00	0.00	0.00	1.47	9,968.85
18	9	0.18	0.06	2,025.03	0.28	0.10	3,297.06	0.10	0.21	1,598.21	0.16	0.33	2,590.79	0.00	0.00	0.00	0.00	0.00	0.00	1.43	9,511.10
19	10	0.17	0.06	1,931.04	0.27	0.09	3,145.05	0.10	0.20	1,524.03	0.16	0.33	2,471.34	0.00	0.00	0.00	0.00	0.00	0.00	1.39	9,071.46
