# Supplementary material for: Cost-effectiveness and budget impact analyses of dengue vaccination in Indonesia
Source: PLoS Negl Trop Dis. 2021 Aug 12;15(8):e0009664. doi: 10.1371/journal.pntd.0009664 (PMC8384188; doi:10.1371/journal.pntd.0009664)
Supplement: S7 Appendix — (PDF) [file pntd.0009664.s007.pdf]

| Outcomes per Cohort | UNDISCOUNTED | | | DISCOUNTED | | |
|---|---|---|---|---|---|---|
| | *Non Vaccine* | *Vaccine* | *Difference* | *Non Vaccine* | *Vaccine* | *Difference* |
| **BURDEN** | **575,885** | **397,890** | **177,996** | **382,416** | **264,217** | **118,199** |
| DF (outpatient) | 295,973 | 204,492 | 91,481 | 196,540 | 135,793 | 60,748 |
| DF (hospitalization) | 23,214 | 16,039 | 7,175 | 15,415 | 10,650 | 4,765 |
| DF (fatal) | 2,649 | 1,830 | 819 | 1,759 | 1,215 | 544 |
| DHF (outpatient) | 170,687 | 117,931 | 52,757 | 113,345 | 78,312 | 35,033 |
| DHF (hospitalization) | 80,548 | 55,652 | 24,896 | 53,488 | 36,956 | 16,532 |
| DHF (fatal) | 2,085 | 1,441 | 645 | 1,385 | 957 | 428 |
| DSS (outpatient) | 0 | 0 | 0 | 0 | 0 | 0 |
| DSS (hospitalization) | 729 | 504 | 225 | 484 | 334 | 150 |
| DSS (fatal) | 0 | 0 | 0 | 0 | 0 | 0 |
| **QALYs LOST** | **275,923.42** | **190,639.85** | **85,283.57** | **184,088.54** | **127,189.68** | **56,898.86** |
| Non-fatal | 38.72 | 26.75 | 11.97 | 25.71 | 17.76 | 7.95 |
| Fatal | 275,884.71 | 190,613.10 | 85,271.61 | 184,062.83 | 127,171.92 | 56,890.91 |

| Outcomes per Cohort | UNDISCOUNTED | | | DISCOUNTED | | |
|---|---|---|---|---|---|---|
| | *Non Vaccine* | *Vaccine* | *Difference* | *Non Vaccine* | *Vaccine* | *Difference* |
| **HEALTHCARE** | | | | | | |
| Vaccine cost | $0 | $441,180,274 | -$441,180,274 | $0 | $334,437,563 | -$334,437,563 |
| Treatment cost | $40,085,623 | $27,695,790 | $12,389,833 | $26,618,814 | $18,391,359 | $8,227,455 |
| Total | $40,085,623 | $468,876,064 | -$428,790,441 | $26,618,814 | $352,828,922 | -$326,210,108 |
| Cost/QALY gained | | | **$5,028** | | | **$5,733** |
| Cost/life saved | | | **$293,323** | | | **$335,696** |
| **PAYER** | | | | | | |
| Vaccine cost | $0 | $441,180,274 | -$441,180,274 | $0 | $334,437,563 | -$334,437,563 |
| Treatment cost | $24,105,129 | $16,654,614 | $7,450,515 | $16,006,984 | $11,059,478 | $4,947,506 |
| Total | $24,105,129 | $457,834,888 | -$433,729,759 | $16,006,984 | $345,497,041 | -$329,490,057 |
| Cost/QALY gained | | | **$5,086** | | | **$5,791** |
| Cost/life saved | | | **$296,701** | | | **$339,071** |

| Level of Severity | | 2020 | 2021 | 2022 | 2023 | 2024 | Total |
|---|---|---|---|---|---|---|---|
| **DF** | outpatient | 5,678 | 21,733 | 31,578 | 53,286 | 60,748 | 173,023 |
| | hospitalization | 445 | 1,705 | 2,477 | 4,179 | 4,765 | 13,570 |
| | death | 51 | 195 | 283 | 477 | 544 | 1,549 |
| **DHF** | outpatient | 3,274 | 12,534 | 18,211 | 30,730 | 35,033 | 99,782 |
| | hospitalization | 1,545 | 5,915 | 8,594 | 14,502 | 16,532 | 47,088 |
| | death | 40 | 153 | 222 | 375 | 428 | 1,219 |
| **DSS** | outpatient | 0 | 0 | 0 | 0 | 0 | 0 |
| | hospitalization | 14 | 54 | 78 | 131 | 150 | 426 |
| | death | 0 | 0 | 0 | 0 | 0 | 0 |

| Perspective | 2020 | 2021 | 2022 | 2023 | 2024 | Total |
|---|---|---|---|---|---|---|
| Healthcare | $769,000 | $2,943,480 | $4,276,821 | $7,216,940 | $8,227,455 | $23,433,695 |
| Payer | $462,431 | $1,770,035 | $2,571,828 | $4,339,842 | $4,947,506 | $14,091,642 |
