# Supplementary material for: Cost-effectiveness and budget impact analyses of dengue vaccination in Indonesia
Source: PLoS Negl Trop Dis. 2021 Aug 12;15(8):e0009664. doi: 10.1371/journal.pntd.0009664 (PMC8384188; doi:10.1371/journal.pntd.0009664)
Supplement: Attachment — Submitted filename: Cover Letter_R.docx [file pntd.0009664.s008.docx]

Dear Editorial Board of PLOS Neglected Tropical Diseases,

By this email, we would like to response reviewers’ comments regarding the proof of our article:

Manuscript number: 260377

Manuscript title: Cost-Effectiveness and Budget Impact Analyses of Dengue Vaccination in Indonesia

**Methods**
Reviewer #1

This is a very relevant study, with methods seeming to have incorporated all relevant aspects of dengue epidemiology and costing. I would like to urge the authors to be bit more extensive on the accuracy and validity of the QALY estimates. It could be considered bit more in the Discussion. I guess there is large uncertainty around them.
Response:

Instead of using secondary data on QALY estimates, we have calculated QALY losses in Indonesia due to dengue infection by delivering a retrospective pre-post questionnaire to 144 patients in 3 cities (Jakarta, Bandung and Yogyakarta), which represented regions with high prevalence of dengue infection in Indonesia. We have added this information now in the methods section (outcome measures).

Reviewer #2

Methods were clearly described. Model and parameters used in the model were clearly described.

1. I would like to know why side-effects of the CYD-TDV vaccine not included in the modelling or in the scenario analysis.

Response:

We have included the side effect of dengue vaccine in the modelling by taking into account the cost of minor side effects that can be potentially resulted, such as localized pain, swelling, fever and aches. We have clarified this better now in the methods section (treatment and vaccination costs).

2. Pre-vaccination screening methods are now recommended by the WHO for dengue vaccine introduction. I would like to see this added to the CEA and BIA, as pre-vaccination screening would be an additional programmatic cost, and this might shift CEA results to not cost-effective.

Response:

A pre-vaccination screening was specifically taken into account in this study, as suggested by the reviewer. We applied a strategy of routine vaccination in which a proportion of children underwent serological screening and vaccination in the event of a positive result would be implemented on their ninth birthday. As its consequence, the intervention coverage (*i.e.,* the proportion of children screened) would represent an upper limit on the proportion of vaccine-eligible children. Seropositivity among 9 years old population (SP_9_) was defined as:

SP_9_ = prior exposure in 9 years old before vaccination (PE_9_) x sensitivity + (1-PE_9_) x (1-specificity).

We applied PE_9_ in Indonesia would be 65.4% (95% CI: 69.1–71.7%) according to the recent study. In addition, we applied a sensitivity of 74.8% (95%CI: 73.0-76.8%) and a specificity of 76.3% (95%CI: 75.2-77.6%) for the diagnosis of dengue. According to these data, we calculated SP_9_ in Indonesia would be 57.12%. We have clarified this better now in the methods section (pre-vaccination screening).

Reviewer #3

As I explain in the overall comments, I think the methods are insufficient for this study.

Response:

We have improved the methods section, as suggested by the reviewer. Firstly, we considered pre-vaccination screening, as recommended by the WHO to minimize the risk of vaccination to seronegative population. Secondly, we took the secondary infection into account. To estimate the epidemiological burden, we applied probabilities of primary and secondary dengue infection in DF, DHF and DSS for outpatient and hospitalization cases by considering the national administrative data and the results of several previous studies. Another improvement that we have conducted is we collected primary data to estimate the QALY loss due to dengue infection.

**Results**
Reviewer #1

Please discuss the potentials of vaccination for other areas than the currently selected regions in Indonesia.

Response:

We have developed an introduction scenario of dengue vaccination in Indonesia by prioritizing provinces with high incidence rate. We targeted number of eligible populations to be vaccinated would be 439,400; 1,681,880; 2,443,740; 4,123,700 and 4,701,100 in a period of 2020-2024. We have clarified this better now in Table 2 (introduction scenario of dengue vaccination in Indonesia).

Reviewer #2

I'm concerned with the use of the so-called WHO criteria for cost-effectiveness threshold based on a countries GDP. The WHO has stated several years ago that GDP per capita thresholds are not recommended. Also, thresholds from 1-3 GDP per capita are very easily achievable. Please suggest an alternative for Indonesia that makes more sense.

Response:

We have presented cost-effectiveness acceptability curves to illustrate about the probability of vaccination program to be cost-effective under various thresholds. Applying a threshold ICER of $3,859 (GDP per capita), the probability for the vaccination program to be cost-effective would be 0% from both perspectives. Applying a threshold ICER of $4,571 (ICER from the healthcare perspective), the probability for the vaccination program to be cost-effective would be 50.5% and 23.1% from the healthcare and payer perspective, respectively. We have added this information now in the fourth paragraph of results section and in the figure 4a.

Reviewer #3

The results are well presented and match their analysis plan. They are clearly presented. Figures are clear, except for figure 3, where the bars are below the text.

Response:

Figure has been improved accordingly.

**Conclusions**
Reviewer #1

Adequately based on the Methods and Results

Reviewer #2

Discussion is good. Strength and weakness of the study detailed.

Reviewer #3

The conclusions are well written and show the public relevance of their study. However, the conclusions are limited by the methodology used to obtain the results (as stated in my overall comments)

Response:

Methods and discussion sections have been improved, as suggested by the reviewer.

**Summary and General Comments**
Reviewer #1: (No Response)

Reviewer #2: (No Response)

Reviewer #3

The manuscript describes a cost-effectiveness analysis of dengue vaccination in Indonesia using a decision tree model. The vaccine being evaluated is the CYD-TDV. The authors focused on a cohort of 9-year-old children followed for 10 years, and assumed implementation of vaccination in 6 provinces every year. Burden of disease was evaluated with a static model of incidence of dengue fever and severe dengue, as well as annual deaths. The cost effectiveness was determined using GDPs thresholds for the Incremental Cost Effectiveness Ratio by QALYs gained. The authors found that vaccination would be cost-effective but not affordable for Indonesia.

The manuscript addresses a very important public health issue, which is to determine the cost-effectiveness of dengue vaccination in an endemic country such as Indonesia. The economic analysis seems sound and the authors presented in a clear manner. However, the methodology used by the authors ignored very important characteristics of dengue transmission and the CYD-TDV vaccine, which should not be ignored in a cost-effectiveness analysis of the implementation of this vaccine.

1. The authors completely omitted any reference to the results from the CYD-TDV trial (e.g., Sridhar et al, 2018) and the safety issues found with the vaccine. The CYD-TDV vaccine was found to elevate the risk of severe disease in post-vaccination infections in vaccines without prior exposure to dengue virus at the time of vaccination. This is a key characteristic of the vaccine and lead to a revision of the WHO recommendation on the use of the vaccine. The WHO now recommends that pre-vaccination screening strategy to vaccinate only seropositive individuals is the preferred option for dengue vaccine implementation (https://www.who.int/immunization/research/development/dengue_q_and_a/en/). Given that the authors do not include this elevated risk in individuals without prior exposure to dengue virus, I think their analysis is incomplete and might overestimate the economic benefits of vaccination, and more importantly they would overestimate the reduction on dengue burden with the vaccine. Many researchers have addressed this issue in cost-effectiveness analyses (Some of them are Flasche et al., 2016, Coudeville et al., 2019, España et al., 2019). Even the reference that the authors cited (Zeng et al., 2018) considers this issue with the vaccine.

Response:

A pre-vaccination screening was specifically taken into account in this study, as suggested by the reviewer. We applied a strategy of routine vaccination in which a proportion of children underwent serological screening and vaccination in the event of a positive result would be implemented on their ninth birthday. As its consequence, the intervention coverage (*i.e.,* the proportion of children screened) would represent an upper limit on the proportion of vaccine-eligible children. Seropositivity among 9 years old population (SP_9_) was defined as:

SP_9_ = prior exposure in 9 years old before vaccination (PE_9_) x sensitivity + (1-PE_9_) x (1-specificity).

We applied PE_9_ in Indonesia would be 65.4% (95% CI: 69.1–71.7%) according to the recent study. In addition, we applied a sensitivity of 74.8% (95%CI: 73.0-76.8%) and a specificity of 76.3% (95%CI: 75.2-77.6%) for the diagnosis of dengue. According to these data, we calculated SP_9_ in Indonesia would be 57.12%. We have clarified this better now in the methods section (pre-vaccination screening).

2. Dengue burden. The authors acknowledge that the static model might not be enough to reproduce the dynamics of transmission of dengue virus in Indonesia. In addition to that, I also think the model might not be sufficient because it does not include primary, secondary, and post-secondary incidence rates. Given the characteristics of the dengue vaccine, these rates are important to determine its impact on the population.

Response:

We have improved the methods section, as suggested by the reviewer. To estimate the epidemiological burden, we applied probabilities of primary and secondary dengue infection in DF, DHF and DSS for outpatient and hospitalization cases by considering the national administrative data and the results of several previous studies. We have clarified this better now in the methods section (disease burden estimates).

We look forward to receive your responses soon regarding our revised article. Thank you very much.

Sincerely,

Auliya A. Suwantika, Ph.D. (corresponding author)
