# Supplementary material for: Cost-effectiveness and budget impact analyses of dengue vaccination in Indonesia
Source: PLoS Negl Trop Dis. 2021 Aug 12;15(8):e0009664. doi: 10.1371/journal.pntd.0009664 (PMC8384188; doi:10.1371/journal.pntd.0009664)
Supplement: Attachment — Submitted filename: Reply to journal.docx [file pntd.0009664.s009.docx]

Dear editors,

The authors have made substantial amendments to the paper but several crucial issues have not been adequately addressed. The authors did not present their changes in their response linked to the location in the manuscript, making it difficult for reviewers.

1. Side-effects of CYD-TDV vaccine – response acceptable
2. Prevaccination screening methods- now added in the CEA. I would like this to be explained in the Introduction. It is important to state that this vaccine has safety concerns among immunologically naïve, as such WHO has recommended prevaccination screening before immunisation.
3. WHO criteria for cost-effectiveness. WHO has denied recommending thresholds based on GDP for cost-effectiveness.
1. Marseille E, Larson B, Kazi DS, et al. Thresholds for the cost–effectiveness of interventions: alternative approaches. *Bulletin of the World Health Organization* 2015;93(2):118-24.
2. Bertram MY, Lauer JA, De Joncheere K, et al. Cost-effectiveness thresholds: pros and cons. *Bulletin of the World Health Organization* 2016;94(12):925-30.
3. 1. Eichler H-G, Kong SX, Gerth WC, et al. Use of cost-effectiveness analysis in health-care resource allocation decision-making: How are cost-effectiveness thresholds expected to emerge? *Value in Health* 2004;7(5):518-28.
4. Newall A, Jit M, Hutubessy R. Are current cost-effectiveness thresholds for low-and middle-income countries useful? Examples from the world of vaccines. *Pharmacoeconomics* 2014;32(6):525-31.
5. Loganathan T, Ng C-W, Lee W-S, et al. Thresholds for decision-making: informing the cost-effectiveness and affordability of rotavirus vaccines in Malaysia. *Health Policy and Planning* 2018;33(2):204-14. doi: 10.1093/heapol/czx166

Please remove references to the WHO threshold from the manuscript, as they are misleading. Please provide a better justification for using GDP based thresholds.

Page 10, Line 160-163:

*The incremental cost-effectiveness ratio (ICER) was evaluated by using the WHO’s criteria on cost-effectiveness of universal immunization according to the GDP per capita, (i) highly cost-effective (less than one GDP per capita); (ii) cost-effective (between 1-3 times GDP per capita); and (iii) cost-ineffective (more than 3 times GDP per capita) [26].*
