# Supplementary material for: Cost-effectiveness and budget impact analyses of dengue vaccination in Indonesia
Source: PLoS Negl Trop Dis. 2021 Aug 12;15(8):e0009664. doi: 10.1371/journal.pntd.0009664 (PMC8384188; doi:10.1371/journal.pntd.0009664)
Supplement: Attachment — Submitted filename: Response to reviewers2.docx [file pntd.0009664.s010.docx]

Dear Editorial Board of PLOS Neglected Tropical Diseases,

By this email, we would like to response reviewers’ comments regarding the proof of our article:

Manuscript number: 260377

Manuscript title: Cost-Effectiveness and Budget Impact Analyses of Dengue Vaccination in Indonesia

The Reviewers believe that some of the issues raised in a previous round of review were addressed. However, there are still a number of other remaining concerns they raised that need to be considered. Specifically:

- Please address how the GDP-based thresholds used in this analysis line up with WHO recommendations and adjust text accordingly (as recommended by Reviewer 2).

Response:
The incremental cost-effectiveness ratio (ICER) was evaluated by using the Commission for Macroeconomics and Health (CMH) thresholds, which were based on the human capital theory and the argument of saving a life year could create market income at least equivalent to the average wage. Interventions with ICER below GDP per capita are highly cost-effective, while those between 1-3 times GDP per capita are cost-effective, and those above 3 times GDP per capita are cost-ineffective. We have clarified this better now in the methods section (cost-effectiveness and budget impact analyses, line 297-302).

- Provide better description of the screening assay and better data points and reference for sensitivity and specificity of the screening assay - the reference and sensitivity/specificity provided are for a classifier for acute dengue, not for measuring past exposure (which I believe would be an IgG ELISA).

## Response: A pre-vaccination screening was specifically taken into account in this study, as recommended by the WHO. We applied a strategy of routine vaccination in which a proportion of children underwent serological screening and vaccination in the event of a positive result would be implemented on their ninth birthday. As its consequence, the intervention coverage (*i.e.,* the proportion of children screened) would represent an upper limit on the proportion of vaccine-eligible children. According to a previous study on the impact of dengue vaccination following screening for prior exposure, seropositivity among 9 years old population (SP_9_) could be defined as:

## *SP_9_ = prior exposure in 9 years old before vaccination (PE_9_) x sensitivity + (1-PE_9_) x (1-specificity).*

## We applied PE_9_ in Indonesia would be 83.1% (95% CI: 77.1–89.0%), according to a previous study by Prayitno *et al*. In addition, we applied a sensitivity of 95.2% (95%CI: 94.2-96.2%) and a specificity of 93.4% (95%CI: 89.6-97.2%) for determining dengue virus serostatus by indirect IgG ELISA with false positivity and negativity of 6.6% and 4.8%, respectively. According to these data, we calculated SP_9_ in Indonesia would be 80.23%. We have clarified this better now in the methods section (pre-vaccination screening, line 168-182).

- Also, please review whether the calculation for adjusting the seroprevalence is correct - I'm not sure I follow the rationale for the PE9*sensitivity + (1-PE9)(1-specificity) calculation. Please review whether this is correct (vs perhaps PE9*specificity + (1-PE9)(1-sensitivity)) or even necessary. Won't the screening assay have some level of false positives and false negatives that need to be considered, rather than adjusting the estimated population prevalence up front?
  Response:

According to a previous study on the impact of dengue vaccination following screening for prior exposure, we calculated seropositivity among 9 years old population (SP_9_) by applying this formula:

## SP_9_ = prior exposure in 9 years old before vaccination (PE_9_) x sensitivity + (1-PE_9_) x (1-specificity).

## We have added this information in the methods section (pre-vaccination screening, line 174-177). In addition, we have taken into account the value of sensitivity and specificity in the sensitivity analysis. We have clarified this better now in the figure 3.

- Along those lines, as one of the Reviewers mentioned, it's probably important to address potential impact of vaccination on individuals with no prior dengue exposure (e.g., false positive on the screen test) in terms of increasing potential for severe disease.

Response:

We did not consider the potential impact of vaccination on individuals with no prior dengue exposure since vaccination could increase the risk of severe dengue in those who have not previously been infected. We have added this information now in the methods section (pre-vaccination screening, line 172-174).

- Provide a clearer explanation of how the 10 year follow-up fits in with the budget analysis.
  Response:

An age-structured decision tree model was developed to assess the cost-effectiveness and the budget impact of dengue vaccination in Indonesia in 2020-2024. To estimate the cost-effectiveness value, a single cohort of 4,710,100 children, started at the age of 9 years old (recommended age for dengue vaccination in Indonesia), was followed-up in a 10-year time horizon within a 1-year analytical cycle. The budget impact analyses was focused on a 5-year period (2020-2024) by considering provinces’ readiness to introduce dengue vaccine and their incidence rate of dengue infection in the last 10 years. We clarified this better now in the methods section (model, line 118-124).

There are a number of other points to consider from the Reviewers below. In addition, there are a few other areas that need to be clarify:

- Line 90: Please provide a reference for the program @Risk.

Response:

A reference for @Risk program has been provided.

- Lines 93-96: Please explain how these DF, DHF, and DSS probabilities were derived, beyond "national administrative data and the results of several previous studies" - how were all of these data sources used and merged together into those values?

Response:

To estimate the epidemiological burden of dengue in Indonesia, we applied probabilities of primary (0.1531, 0.2306 and 0.0015 for DF, DHF and DSS, respectively) and secondary dengue infection (0.2449, 0.3674 and 0.0025 for DF, DHF and DSS, respectively), outpatient (0.6800, 0.2610 and 0.0000 for DF, DHF and DSS, respectively) and hospitalization cases (0.3200, 0.7390 and 1.0000 for DF, DHF and DSS, respectively), according to the national administrative data and the results of several previous studies. For DF outpatient cases, we estimated the probabilities of primary and secondary infections would be 0.1041 (0.1531*0.6800) and 0.1655 (0.2449*0.6800), respectively. For DF hospitalization cases, we estimated the probabilities of primary and secondary infections would be 0.0490 (0.1531*0.3200) and 0.0784 (0.2449*0.3200), respectively. We also estimated the probabilities of primary and secondary infections for DHF outpatient cases would be 0.0602 (0.2306*0.2610) and 0.0959 (0.3674*0.2610), respectively. For DHF hospitalization cases, the probabilities of primary and secondary infections would be 0.1704 (0.2306*0.7390) and 0.2715 (0.3674*0.7390), respectively. In contrast with other cases, we estimated there would not be primary and secondary infections for DSS outpatient cases. For DSS hospitalization cases, we estimated the probabilities of primary and secondary infections would be 0.0015 (0.0015*1.0000) and 0.0025 (0.0025*1.0000), respectively. We have clarified this better now in the methods section (disease burden estimates, line 140-158).

- Line 225: The sentence states that vaccination could avert 84,155 cases annually, but I don't see where that calculation comes from.

Response:

This study confirmed that a nationwide dengue vaccination appears to be one of promising interventions to prevent DENV infection by showing potential benefits on reducing DF, DHF and DSS by 188,142, 148,089 and 426 cases, respectively. We have clarified this better now in the first paragraph of discussion section (line 595).

- Line 254: The sentence seems to be incomplete: "...the difficulty in obtaining." - obtaining what?

Response:

The key challenge to conduct economic evaluation studies in low- and middle-income countries is the difficulty in obtaining local data. We have revised the sentence accordingly (line 641).

- Line 270: "The static model tends to over-estimate the cost-effectiveness results due to the inability to incorporate the herd effect. In particular, if we took herd effect into account, there would be an even more favourable cost-effectiveness." I'm not sure I follow - sounds like the model would overestimate cost-effectiveness, but then you are saying that the change would be more favourable?
  Response:

We have rewritten the sentence (line 656-657) to be much clearer for the reader, as suggested by the reviewer. We found several limitations in this study. The first and main limitation is the use of the static model instead of the dynamic model due to the lack of local data on herd effect. If we took herd effect into account, the cost-effectiveness value would be more favourable.

Reviewer #2

- Side-effects of CYD-TDV vaccine – response acceptable.

Response:

We have considered side effects in our cost estimation. Thank you very much for your comment.

- Pre-vaccination screening methods- now added in the CEA. I would like this to be explained in the Introduction. It is important to state that this vaccine has safety concerns among immunologically naïve, as such WHO has recommended pre-vaccination screening before immunization.

Response:

Vaccine-preventable diseases has been estimated to be a cost-effective strategy since 1993. For dengue vaccination, it has been expected to be a highly cost-effective or even cost-saving intervention in ten endemic countries. On the other hand, a recent study confirmed an elevated of severe disease in vaccinees with no prior DENV exposure, which made an end to the implementation of dengue vaccine in the Philippines. Therefore, the World Health Organization (WHO) recommended countries to integrate a pre-vaccination screening in dengue vaccination program. We have added this information now in the introduction section (line 85-91), as suggested by the reviewer.

- WHO criteria for cost-effectiveness. WHO has denied recommending thresholds based on GDP for cost-effectiveness.
  1. Marseille E, Larson B, Kazi DS, et al. Thresholds for the cost–effectiveness of interventions: alternative approaches. Bulletin of the World Health Organization 2015;93(2):118-24.
  2. Bertram MY, Lauer JA, De Joncheere K, et al. Cost-effectiveness thresholds: pros and cons. Bulletin of the World Health Organization 2016;94(12):925-30.
  3. 1. Eichler H-G, Kong SX, Gerth WC, et al. Use of cost-effectiveness analysis in health-care resource allocation decision-making: How are cost-effectiveness thresholds expected to emerge? Value in Health 2004;7(5):518-28.
  4. Newall A, Jit M, Hutubessy R. Are current cost-effectiveness thresholds for low-and middle-income countries useful? Examples from the world of vaccines. Pharmacoeconomics 2014;32(6):525-31.
  5. Loganathan T, Ng C-W, Lee W-S, et al. Thresholds for decision-making: informing the cost-effectiveness and affordability of rotavirus vaccines in Malaysia. Health Policy and Planning 2018;33(2):204-14. doi: 10.1093/heapol/czx166
  Response:

The incremental cost-effectiveness ratio (ICER) was evaluated by using the Commission for Macroeconomics and Health (CMH) thresholds, which were based on the human capital theory and the argument of saving a life year could create market income at least equivalent to the average wage. We have clarified this better now in the methods section (cost-effectiveness and budget impact analyses, line 297-300).

- Please remove references to the WHO threshold from the manuscript, as they are misleading. Please provide a better justification for using GDP based thresholds.

Page 10, Line 160-163:
The incremental cost-effectiveness ratio (ICER) was evaluated by using the WHO’s criteria on cost-effectiveness of universal immunization according to the GDP per capita, (i) highly cost-effective (less than one GDP per capita); (ii) cost-effective (between 1-3 times GDP per capita); and (iii) cost-ineffective (more than 3 times GDP per capita) [26].
Interventions with ICER below GDP per capita are highly cost-effective, while those between 1-3 times GDP per capita are cost-effective, and those above 3 times GDP per capita are cost-ineffective.

Response:

References has been removed and a better justification for using GDP based thresholds has been provided in the methods section (cost-effectiveness and budget impact analyses, line 300). In addition, we have added information about the incremental cost-effectiveness ratio (ICER) that was evaluated by using the Commission for Macroeconomics and Health (CMH) thresholds, which were based on the human capital theory and the argument of saving a life year could create market income at least equivalent to the average wage. Interventions with ICER below GDP per capita are highly cost-effective, while those between 1-3 times GDP per capita are cost-effective, and those above 3 times GDP per capita are cost-ineffective. We have clarified this better now in line 297-302.

Reviewer #3

The revision of this manuscript attempted to address my main concerns from the first version. I appreciate the authors' efforts. However, there are relevant points that I consider need to be addressed. My main concern is the implementation of the pre-vaccination screening strategy.

- Sensitivity and specificity of the tests used to determine previous exposure to DENV. The authors describe SP9 as the seropositivity in 9-year-olds, i.e., the proportion of children who would be eligible for vaccination. However, the authors used sensitivity and specificity values of dengue diagnosis, which only detects active DENV infection. Sensitivity and specificity values for pre-vaccination screening should refer to tests to determine previous infection.

Response:

## A pre-vaccination screening was specifically taken into account in this study, as recommended by the WHO. We applied a strategy of routine vaccination in which a proportion of children underwent serological screening and vaccination in the event of a positive result would be implemented on their ninth birthday. As its consequence, the intervention coverage (*i.e.,* the proportion of children screened) would represent an upper limit on the proportion of vaccine-eligible children. According to a previous study on the impact of dengue vaccination following screening for prior exposure, seropositivity among 9 years old population (SP_9_) could be defined as:

## *SP_9_ = prior exposure in 9 years old before vaccination (PE_9_) x sensitivity + (1-PE_9_) x (1-specificity).*

## We applied PE_9_ in Indonesia would be 83.1% (95% CI: 77.1–89.0%), according to a previous study by Prayitno *et al*. In addition, we applied a sensitivity of 95.2% (95%CI: 94.2-96.2%) and a specificity of 93.4% (95%CI: 89.6-97.2%) for determining dengue virus serostatus by indirect IgG ELISA with false positivity and negativity of 6.6% and 4.8%, respectively. According to these data, we calculated SP_9_ in Indonesia would be 80.23%. We have clarified this better now in the methods section (pre-vaccination screening, line 168-182)

- The model omits the elevated risk of severe dengue on vaccinated individuals without previous exposure to dengue virus. Specifically, the decision tree should allow for screening and vaccination of truly seropositive individuals (sensitivity * PE) and truly negative individuals ((1 - specificity) * (1 - PE)).

Response:

We only considered the potential impact of vaccination on individuals with prior dengue exposure since vaccination could increase the risk of severe dengue in those who have not previously been infected. We have added this information now in the methods section (pre-vaccination screening, line 172-174).

- The results about disease rates and cost-effectiveness should include the elevated rates of hospitalization of individuals without previous exposure to DENV.

Response:

We have the same response with the previous question. In this study, we did not consider the potential impact of vaccination on individuals with no prior dengue exposure since vaccination could increase the risk of severe dengue in those who have not previously been infected. We have added this information now in the methods section (pre-vaccination screening, line 172-174). We also have highlighted the main limitation of our study is the use of the static model, which could not take the herd effect into account (line 678-679).

- The authors should discuss the characteristics of the vaccine (CYD-TDV) and its implications for implementation. Only the Philippines and Brazil have attempted to include the vaccine in their vaccination programs. However, due to the negative effects of CYD-TDV, those vaccination strategies were halted. In the manuscript, it's unclear the reason for a pre-vaccination screening strategy, which is the elevated risk in individuals without previous exposure to DENV.

Response:

Vaccine-preventable diseases has been estimated to be a cost-effective strategy since 1993. For dengue vaccination, it has been expected to be a highly cost-effective or even cost-saving intervention in ten endemic countries. On the other hand, a recent study confirmed an elevated of severe disease in vaccinees with no prior DENV exposure, which made an end to the implementation of dengue vaccine in the Philippines. Therefore, the World Health Organization (WHO) recommended countries to integrate a pre-vaccination screening in dengue vaccination program. We have clarified this better now in the introduction section (line 86-91).

Other concerns:

- Line 49,52: "dengue infection" should be "dengue virus infection". In general, I would recommend the authors to double check their use of dengue and dengue virus. Dengue refers to the disease, and dengue virus (DENV) to the virus.

Response:

Revisions have been made accordingly.

- Lines 64-65: "dengue vaccination has been confirmed to be a cost-effective strategy for dengue control". I don't understand that claim. Dengue vaccine was not available in 1993, how can it be confirmed to be cost-effective? Also, given the unexpected elevated risk on seronegative individuals from CYD-TDV, I would recommend to only reference recent studies that take into account the specific characteristics of CYD-TDV for any claims on cost-effectiveness.

Response:

Vaccine-preventable diseases has been estimated to be a cost-effective strategy since 1993. For dengue vaccination, it has been expected to be a highly cost-effective or even cost-saving intervention, according to the recent study on cost-effectiveness of dengue vaccination in ten endemic countries. We have clarified this better now in the introduction section (line 85-88)

- Line 65: "it has been confirmed". Similar to my previous comment. I disagree, it's expected to be cost-effective, but it's not confirmed.

Response:

For dengue vaccination, it has been expected to be highly cost-effective or even cost-saving, according to the recent study on cost-effectiveness of dengue vaccination in ten endemic countries. We have clarified this in line 85-88.

- Line 68-69: "incidence and mortality rates due to dengue infection in Indonesia are relatively high." Please add a reference and numbers to support that statement.

Response:

A reference has been added to support that statement in line 108.

- Lines 68-73: This paragraph speculates the reasons to not implement the vaccine, but it ignores the situation in other countries. To my knowledge, the vaccine has not been implemented in any other country after it was retracted from the Philippines. Other than the economic constraints of Indonesia to implement the vaccine, are there any other reasons that other countries, and Indonesia, have not implemented the vaccine?

Response:

Up to now, there is only one available dengue vaccine (CYD-TDV) that has been marketed in Indonesia since 2016. Despite the fact that the incidence and mortality rates due to DENV infection in Indonesia are relatively high, dengue vaccination has not yet been included into the national immunization program (NIP). This situation might be caused by the lack of economic evaluation studies, which was based on country specific data. In particular, the recommendation of pre-vaccination screening might affect the favourable cost-effectiveness value of dengue vaccination to be implemented in Indonesia. Hence, this study aimed to analyse the cost-effectiveness and the budget impact of dengue vaccination in Indonesia by taking the potential of pre-vaccination screening into account. We have added this information in the introduction section (line 92-114)

- The authors should elaborate on the characteristics of CYD-TDV and why the WHO recommends pre-vaccination screening strategies.

Response:

A recent study confirmed an elevated of severe disease in vaccinees with no prior DENV exposure, which made an end to the implementation of dengue vaccine in the Philippines. Therefore, the World Health Organization (WHO) recommended countries to integrate a pre-vaccination screening in dengue vaccination program. We have clarified this better now in the introduction section (line 88-91)

- It's not clear to me why the budget analysis is from 2020-2024 whereas the cohort is followed by 10 years.

Response:

An age-structured decision tree model was developed to assess the cost-effectiveness and the budget impact of dengue vaccination in Indonesia in 2020-2024. To estimate the cost-effectiveness value, a single cohort of 4,710,100 children, started at the age of 9 years old (recommended age for dengue vaccination in Indonesia), was followed-up in a 10-year time horizon within a 1-year analytical cycle. The budget impact analyses was focused on a 5-year period (2020-2024) by considering provinces’ readiness to introduce dengue vaccine and their incidence rate of dengue infection in the last 10 years. We clarified this better now in the methods section (model, line 118-124)

- Line 82: What's the incidence rate in the last 10 years?

Response:

We considered provinces’ readiness to introduce dengue vaccine and their incidence rate of DENV infection in the last 10 years to analyse the budget impact of vaccination program in a 5-year period (2020-2024). We clarified this better now in the methods section (model, line 122-124)

- Lines 96-100: I don't understand how the authors estimated these probabilities.

Response:

To estimate the epidemiological burden of dengue in Indonesia, we applied probabilities of primary (0.1531, 0.2306 and 0.0015 for DF, DHF and DSS, respectively) and secondary infection (0.2449, 0.3674 and 0.0025 for DF, DHF and DSS, respectively), outpatient (0.6800, 0.2610 and 0.0000 for DF, DHF and DSS, respectively) and hospitalization cases (0.3200, 0.7390 and 1.0000 for DF, DHF and DSS, respectively), according to the national administrative data and the results of several previous studies. For DF outpatient cases, we estimated the probabilities of primary and secondary infections would be 0.1041 (0.1531*0.6800) and 0.1655 (0.2449*0.6800), respectively. The similar approach was applied to estimate the probabilities of primary and secondary infections in DF hospitalization, DHF outpatient, DHF hospitalization, DSS outpatient and DSS hospitalization cases. We have clarified this better now in the methods section (disease burden estimates, line 140-158).

- In general, the clinical manifestations of dengue are grouped into Dengue Fever and Sever Dengue (https://www.ncbi.nlm.nih.gov/pmc/articles/PMC3202316/). It's unclear why the authors decide to use the classification of Dengue Haemorrhagic Fever and Dengue Shock Syndrome.

Response:

We divided the manifestations of DENV infection range from asymptomatic infection or dengue fever (DF), to the more life-threatening forms, dengue haemorrhagic fever (DHF) and dengue shock syndrome (DSS), according to the local study on the epidemiology, virology and clinical findings of DENV infections in Indonesian. We have clarified this better now in line 76-78.

- Line 120: The sensitivity and specificity should be to detect previous exposure to DENV not current infection. There should also be a sensitivity analysis over these values.

Response:

We have considered the sensitivity and specificity in the sensitivity analysis. We have presented this in the figure 3.

- Line 123 - 131: I'm not sure the authors addressed my concerns on the elevated risk for vaccinated seronegative individuals. As it's presented in line 117 (From reference 16), there is a proportion of children without prior exposure to dengue virus who are vaccinated, i.e., (1-PE9)x(1-specificity). These children would have an elevated risk of experiencing severe dengue, which should be included in the costs of the vaccination program. I do not see that estimate in the manuscript.

Response:

We did not consider the potential impact of vaccination on individuals with no prior dengue exposure since vaccination could increase the risk of severe dengue in those who have not previously been infected. We have added this information now in the methods section (pre-vaccination screening, line 172-174).

- Would like to split the results in terms of cases averted into: cases averted by vaccination of previously exposed and the additional cases caused by vaccination of non-previously exposed individuals.

Response:

We have the same response with the previous question. We did not consider the potential impact of vaccination on individuals with no prior dengue exposure since vaccination could increase the risk of severe dengue in those who have not previously been infected. We have added this information now in the methods section (pre-vaccination screening, line 172-174).

- Where did the authors obtained the $10 cost for pre-vaccination screening?

Response:

A vaccine price per dose of $20 and pre-vaccination screening cost of $10 were applied from a study by Zeng *et al.*, which estimated the cost-effectiveness of dengue vaccination in 10 endemic countries, including Indonesia. We have clarified this better now in the methods section (treatment and vaccination costs, line 265-267).

- QALYs calculation should include the cost of additional hospitalizations caused by vaccination of non-exposed individuals.

Response:

We have the same response with the previous question. We did not consider the potential impact of vaccination on individuals with no prior dengue exposure since vaccination could increase the risk of severe dengue in those who have not previously been infected. We have added this information now in the methods section (pre-vaccination screening, line 172-174).

We look forward to receive your responses soon regarding our revised article. Thank you very much.

Sincerely,

Auliya A. Suwantika, Ph.D. (corresponding author)
